# Optimizing nutrient use efficiency, productivity, energetics, and economics of red cabbage following mineral fertilization and biopriming with compatible rhizosphere microbes

**DOI:** 10.1038/s41598-021-95092-6

**Published:** 2021-08-03

**Authors:** Deepranjan Sarkar, Ardith Sankar, O. Siva Devika, Sonam Singh, Shikha, Manoj Parihar, Amitava Rakshit, R. Z. Sayyed, Abdul Gafur, Mohammad Javed Ansari, Subhan Danish, Shah Fahad, Rahul Datta

**Affiliations:** 1 Department of Soil Science and Agricultural Chemistry, Institute of Agricultural Sciences, Banaras Hindu University, Uttar Pradesh, Varanasi, 221005 India; 2 Department of Agronomy, Institute of Agricultural Sciences, Banaras Hindu University, Uttar Pradesh, Varanasi, 221005 India; 3 Krishi Vigyan Kendra, Ranichauri, Veer Chandra Singh Garhwali Uttarakhand University of Horticulture and Forestry, Tehri Garhwal, 249199 Uttarakhand India; 4 Crop Production Division, ICAR-Vivekananda Parvatiya Krishi Anusandhan Sansthan, Almora, 263601 Uttarakhand India; 5 Department of Microbiology, PSGVP Mandal’s, Arts, Science & Commerce College, 425409, Shahada, Maharashtra India; 6 Sinarmas Forestry Corporate Research and Development, Perawang, 28772 Indonesia; 7 Department of Botany, Hindu College Moradabad (Mahatma Jyotiba Phule Rohilkhand Univesity Bareilly), Moradabad, Uttar Pradesh 244001 India; 8 Department of Soil Science, Faculty of Agricultural Sciences and Technology, Bahauddin Zakariya University, Multan, 60800 Pakistan; 9 Department of Agronomy, The University of Haripur, Haripur, 22620 Pakistan; 10 Department of Geology and Pedology, Faculty of Forestry and Wood Technology, Mendel University in Brno, Zemedelska1, 61300 Brno, Czech Republic

**Keywords:** Plant sciences, Plant symbiosis

## Abstract

Conventional agricultural practices and rising energy crisis create a question about the sustainability of the present-day food production system. Nutrient exhaustive crops can have a severe impact on native soil fertility by causing nutrient mining. In this backdrop, we conducted a comprehensive assessment of bio-priming intervention in red cabbage production considering nutrient uptake, the annual change in soil fertility, nutrient use efficiency, energy budgeting, and economic benefits for its sustainable intensification, among resource-poor farmers of Middle Gangetic Plains. The compatible microbial agents used in the study include *Trichoderma harzianum*, *Pseudomonas fluorescens*, and *Bacillus subtilis*. Field assays (2016–2017 and 2017–2018) of the present study revealed supplementing 75% of recommended NPK fertilizer with dual inoculation of *T*. *harzianum* and *P*. *fluorescens* increased macronutrient uptake (N, P, and K), root length, heading percentage, head diameter, head weight, and the total weight of red cabbage along with a positive annual change in soil organic carbon. Maximum positive annual change in available N and available P was recorded under 75% RDF + *P*. *fluorescens* + *B*. *subtilis* and 75% RDF + *T*. *harzianum* + *B*. *subtilis*, respectively. Bio-primed plants were also higher in terms of growth and nutrient use efficiency (agronomic efficiency, physiological efficiency, apparent recovery efficiency, partial factor productivity). Energy output (26,370 and 26,630 MJ ha^−1^), energy balance (13,643 and 13,903 MJ ha^−1^), maximum gross return (US $ 16,030 and 13,877 ha^−1^), and net return (US $ 15,966 and 13,813 ha^−1^) were considerably higher in *T*. *harzianum,* and *P*. *fluorescens* treated plants. The results suggest the significance of the bio-priming approach under existing integrated nutrient management strategies and the role of dual inoculations in producing synergistic effects on plant growth and maintaining the soil, food, and energy nexus.

## Introduction

India has attained ‘self-sufficiency’ in food grain production, but some factors like declining nutrient use efficiency, low crop response ratio, negative soil nutrient balance, etc., have become major concerns in Indian agriculture. The accomplishment of the Green Revolution has left some drastic effects on our motherland soil. As we fulfilled our target of increased food grain production, there are indications of declining partial and total factor productivity, and the sole cause has been attributed to diminishing native soil fertility ^1^. Therefore, the dwindling natural resource base has raised sustainability concerns and integrated resource management in the present agricultural system. Moreover, climate change has forced us to adopt crop management practices more scientifically to sustainable land use and land cover^2^.

Beneficial microorganisms play an important role in achieving agro-environmental sustainability. However, the abundance and robustness of microbes are dependent upon smart delivery systems^3^. Bio-priming is one possible method to apply plant growth-promoting microorganisms in the soil–plant system and improve the nutrient use efficiency of agroecosystems^4,5^. Primers' selection is an essential step in biological seed enhancements as priming agents' growth-promoting abilities are highly specific to certain species, cultivars, or genotypes of crops^6^. A microbial consortium treatment can also be used for getting better results after checking the compatibility of the microbes^7^. This will promote synergistic interactions among the microbial population^8^. The literature of current decades reveals that beneficial microorganisms and their interactions with host plants harbor broad prospects in sustainable agriculture's persistence. Their influence in the rhizosphere ecosystem compels researchers to investigate their ecology, diversity, and activity, contributing most to soil health and plant fitness. The rhizosphere is chosen because it is the hub of a complex web of interactions regulated by the system's enormous energy flux, releasing about 20–50% of plant photosynthates from the roots^9,10^. Thus, strong documentation is required on how species richness affects the ecological functions of agroecosystems. Understanding the microbial mechanisms influencing plant productivity is key to augment plant growth and boost the processes within the soil system. In general, the direct growth-promoting mechanisms used by them can be listed as (i) nitrogen (N) fixation; (ii) nutrient solubilization (phosphorus (P), potassium (K), etc.); (iii) production of phytohormones, e.g., auxins (indole-3-acetic acid, indole-3-butyric acid), cytokinins, gibberellins, abscisic acid, and ethylene; (iv) iron sequestration by siderophore production; (v) regulation of plant hormones, e.g., 1-aminocyclopropane-1-carboxylate (ACC) deaminase decreases ethylene levels; and the indirect ones include (i) induction of systemic resistance against plant pathogens through antagonism with the production of inhibitory compounds or substances; (ii) suppression of phytopathogens with the synthesis of hydrolytic enzymes (glucanases, chitinases, proteases, and lipases), siderophores, antibiotics, and cyanide^11–14^.

The application of fertilizers for crop production is increasing rapidly. However, the average N recovery efficiency and P recovery efficiency are less than 50% and 20%, respectively^15^. Some recent studies showed improved nutrient use efficiency due to microbial inoculation of plants. Soils treated with 50% organic and inorganic N fertilizers and the application of *Paraburkholderia* sp. augmented the growth of kikuyu grass and N use efficiency compared to 100% N fertilizer treatment^16^. Pereira et al.^17^ reported improved N use efficiency and P use efficiency of maize plants treated with *Cupriavidus necator* and *Pseudomonas fluorescens*. However, these experiments were performed under pot conditions.

The modern agricultural system depends on energy inputs, viz., fossil fuels, fertilizers, pesticides, electricity, etc. that affect our ecosystem's health and increase greenhouse gas emissions^18^. The extent of energy consumption is evident from the fact that the production of nitrogen (N), phosphorus (P), and potassium (K) fertilizers require approximately 60.6, 11.1, and 6.7 MJ kg^−1^ energy, respectively^19,20^. However, very few studies have evaluated the energetics of crop production involving microbial application, mainly based on controlled conditions. Mihov and Tringovska^21^ evaluated the energy use efficiency of greenhouse tomato production. Pal and Singh^22^ examined the relationship of energy inputs and crop yield in greenhouse okra production.

With growing agro-environmental challenges, we need to look for new, nutritive, and versatile crops to make agriculture sustainable and profitable. Red cabbage is an excellent source of minerals, vitamins, antioxidants, anthocyanin, glucosinolates, and phenolic compounds^23,24^. Therefore, our diet is often chosen as salads, microgreens, leafy vegetables, and health-improving supplements. In addition, supermarkets will provide assured economic returns to farmers. Hence, the present study's primary objective was to investigate the effect of bio-priming and mineral fertilization on red cabbage growth, nutrient uptake, the annual change in soil fertility, nutrient use efficiency, energy usage, and economic returns under field conditions.

## Results and discussion

### Macronutrient uptake

Total uptake of N, P, and K by red cabbage was partitioned into head and stalk. The head's highest N uptake was registered in plots receiving 75% RDF + *T*. *harzianum* + *P*. *fluorescens* (T_6_) during both the years (Table S1). It showed 29% (1^st^ year) and 24% (2^nd^ year) increments over 100% RDF (T_2_). Similarly, a significant increase in total uptake of N was also observed in T_6_ treatment. The total N uptake varied from 21.53 to 70.21 kg ha^−1^ in the first year and 19.56–69.96 kg ha^−1^ in the second year. Compared with single-priming and consortium treatments, N total uptake increased by 12–35% with the co-application of *T*. *harzianum* and *P*. *fluorescens*. The uptake of the other two dual bio-priming treatments, viz., T_7_ (75% RDF + *P*. *fluorescens* + *B*. *subtilis*) and T_8_ (75% RDF + *T*. *harzianum* + *B*. *subtilis*) were at par with T_2_ (100% RDF). Phosphorus uptake by red cabbage was also influenced by 75% RDF + *T*. *harzianum* + *P*. *fluorescens* (T_6_), but it was at par with 75% RDF + *P*. *fluorescens* + *B*. *subtilis* (T_7_). In the head, P uptake ranged from 1.28 to 7.36 kg ha^−1^ in the first year and 0.80–7.52 kg ha^−1^ in the second year (Table S2). During the second year, the head uptake of plants under 75% RDF + *P*. *fluorescens* (T_4_) was higher than 75% RDF + *T*. *harzianum* + *B*. *subtilis* (T_8_). The application of fertilizers and bio-priming agents did not significantly affect the P uptake by stalk during the study years. The total P uptake varied from 3.69 to 10.99 kg ha^−1^ in the first year and 3.44–11.29 kg ha^−1^ in the second year. Sole application of *T*. *harzianum* and *B*. *subtilis* and a triple consortium of *T*. *harzianum*, *P*. *fluorescens*, and *B*. *subtilis* showed statistically similar P uptake. Except for control, the uptake was found to increase in all the treatments during the second year over 1^st^ year. In the case of K, a significant increase in uptake (head and total) was recorded in T_6_ treatment (Table S3). The magnitude of the increase due to this treatment over 100% RDF (T_2_) was 31% (head) and 21% (total). However, applying 100% chemical fertilizers and combined use of chemical fertilizers and bio-agents did not significantly differ in the uptake of macronutrients by the stalk. In general, the K uptake varied from 8.25 to 57.35 kg ha^−1^ for the head and 16.64–22.15 kg ha^−1^ for the stalk. Stalk K uptake was highest (22.15 kg ha^−1^) in the first year with 75% RDF + *B*. *subtilis* (T_5_). The total K uptake ranged from 29.03 to 78.20 kg ha^−1^ in the first year and 26.08–78.51 kg ha^−1^ in the second year. During the second year, the total K uptake of plants under 100% RDF was par with single- and triple-priming treatments. Among single- and triple-priming treatments, the highest total K uptake was observed with the application of 75% RDF + *P*. *fluorescens* (T_4_) during both years. The lowest N, P, and K uptake were recorded from the control (T_1_) plots. On average, red cabbage removed macronutrients in the order of K (64.32 kg ha^−1^) > N (55.05 kg ha^−1^) > P (8.91 kg ha^−1^). The higher nutrient uptake in the integrated application of chemical fertilizers and bio-agents is explained by developing proliferous root systems in bio-primed plants and increased microbial activity in the soil, which helped mineralize nutrients maintaining the soil solution greater assimilation in plants. Several workers^25–31^ reported increased nutrient uptake in crops due to the combined application of chemical fertilizers (reduced level) and biofertilizers.

### Annual change in organic carbon (OC) and available N, P, and K

**Figure 1:**
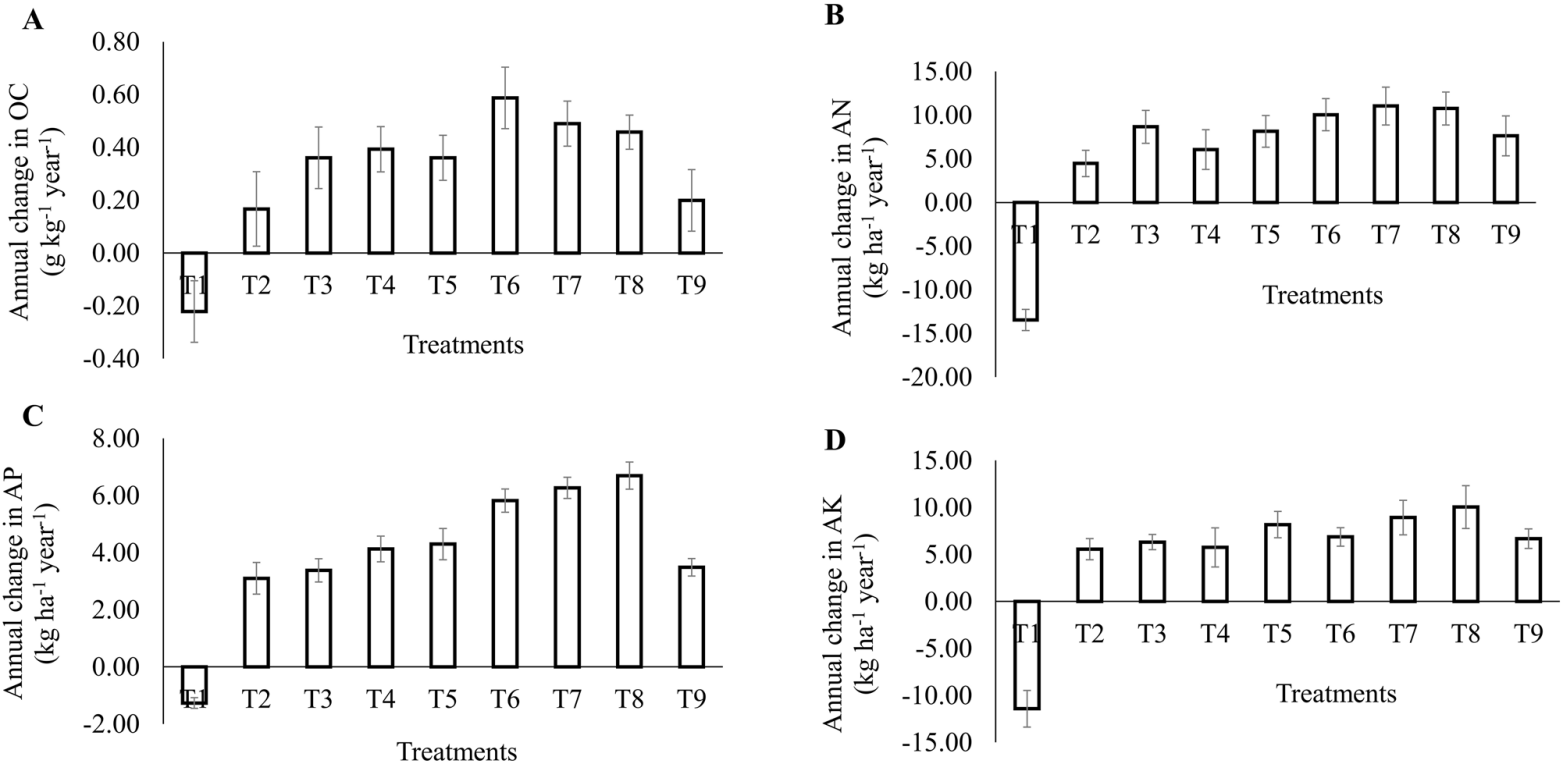
Annual change in organic carbon (**A**), available N (**B**), available P (**C**), and available K (**D**) as affected by bio-priming and mineral fertilization. Error bars indicate mean ± SE (*n* = 3). Treatments: T_1_: Absolute control N:P_2_O_5_:K_2_O @ 0:0:0 kg ha^−1^; T_2_: RDF of N:P_2_O_5_:K_2_O @ 120:60:60 kg ha^−1^; T_3_: 75% RDF + *Trichoderma harzianum*; T_4_: 75% RDF + *Pseudomonas fluorescens*; T_5_: 75% RDF + *Bacillus subtilis*; T_6_: 75% RDF + *T*. *harzianum* + *P*. *fluorescens*; T_7_: 75% RDF + *P*. *fluorescens* + *B*. *subtilis*; T_8_: 75% RDF + *T*. *harzianum* + *B*. *subtilis*; T_9_: 75% RDF + *T*. *harzianum* + *P*. *fluorescens* + *B*. *subtilis.*

A positive annual change in OC (Fig. 1) was noted for all the treatments (T_2_-T_9_) except in control plots (T_1_) which showed a negative change of − 0.22 g kg^−1^ year^−1^. The highest positive change was documented under 75% RDF + *T*. *harzianum* + *P*. *fluorescens* (T_6_, 0.59 g kg^−1^ year^−1^) followed by 75% RDF + *P*. *fluorescens* + *B*. *subtilis* (T_7_, 0.49 g kg^−1^ year^−1^), and the lowest was recorded under 100% chemical fertilization (T_2_) being 0.17 g kg^−1^ year^−1^ which was at par with 75% RDF + *T*. *harzianum* + *P*. *fluorescens* + *B*. *subtilis* (T_9_, 0.20 g kg^−1^ year^−1^). In the case of annual change of available N, P, and K, similar trends were noticed as OC (Fig. 1). However, the highest and lowest changes varied. Application of 75% RDF + *P*. *fluorescens* + *B*. *subtilis* (T_7_) and 75% RDF + *T*. *harzianum* + *B*. *subtilis* (T_8_) demonstrated (Fig. 1) higher positive changes in available N (11.06 and 10.06 kg ha^−1^ year^−1^, respectively), while 100% RDF (T_2_) showed the lowest change (4.47 kg ha^−1^ year^−1^). The control plots (T_1_) showed a significant negative annual change in available N (− 13.46 kg ha^−1^ year^−1^) after crop harvest. The T_8_ treatment showed the maximum positive annual change (6.70 kg ha^−1^ year^−1^) followed by T_7_ (6.27 kg ha^−1^ year^−1^) for available P (Fig. 1). However, the least positive change in available P was evident in T_2_ (3.10 kg ha^−1^ year^−1^), which was at par with the treatment T_9_ (3.48 kg ha^−1^ year^−1^). A negative change of − 1.27 kg ha^−1^ year^−1^ in available P was observed in T_1_ plots. There were no significant positive annual changes observed between the treatments for available K (Fig. 1). However, the T_8_ treatment presented the highest positive annual change value of 10.04 kg ha^−1^ year^−1^, followed by T_7_ (8.92 kg ha^−1^ year^−1^); the rest of the treatments (T_2_, T_3_, T_4_, T_5_, T_6_, and T_9_) documented at par values of the same. The T_1_ plots recorded a significant negative annual change in available K (− 11.43 kg ha^−1^ year^−1^).

Conclusively, due to no supplement of fertilizer nutrients and/or biofertilizers, the control plots exhibited a negative annual change in all the studied cases demonstrating the loss/mining of nutrients. However, where both are supplied, a positive change is noticed, indicating annual enrichment of the respected soil attributes over control. In this study, T_8_ and T_7_ treatments performed better in enhancing available N, P, and K except in OC where T_6_ treatment presented a significant positive result over these two, and the rest of the treatments remained at par with positive change. Furthermore, Kaur and Reddy^32^ reported improved soil fertility using mineral fertilizer and biofertilizer (*Pseudomonas plecoglossicida* and *Pantoea cypripedii*). Similarly, the integrated application of NPK fertilizers and organic amendments (green manure + *Pseudomonas putida* + *Azotobacter chroococcum*) recorded a higher OC value and available N and K over only NPK fertilizer application^33^.

### Nitrogen use efficiency

Optimization of fertilizer for achieving higher input use efficiency without hindering the economic yield is a crucial issue in agriculture. The extent of crop utilization to applied N was analyzed through different parameters of N use efficiency. Agronomic efficiency (AE_N_) varied from 9.48 to 19.38 kg of head kg^−1^ of N applied (Table 1). During the study period of the field experiment, the mean value of AE_N_ increased from 12.95 kg of head kg^−1^ of N applied in 2016–2017 to 15.50 kg of head kg^−1^ of N applied in 2017–2018. The application of 75% RDF + *T*. *harzianum* + *P*. *fluorescens* (T6) increased AEN by 84% and 75% over 100% RDF (T2) during the first and second years. Compared to T_6_, the application of 75% RDF + *T*. *harzianum* + *P*. *fluorescens* + *B*. *subtilis* (T_9_) significantly reduced the AE_N_ by 59% and 39% in the first and second years respectively. Application of triple consortium also recorded low AE_N_ in comparison to single-species bio-priming and dual microbial consortium treatments. Seedling inoculation with biofertilizers along with 75% RDF + vermicompost demonstrated similar results in cabbage^34^.

**Table 1:** Nitrogen use efficiency of red cabbage as influenced by bio-priming and fertilisation.

Treatments	N use efficiency							
	Agronomic efficiency (kg kg^−1^)		Physiological efficiency (kg kg^−1^)		Apparent recovery efficiency (%)		Partial factor productivity (kg kg^−1^)	
	2016–2017	2017–2018	2016–20	2017–2018	2016–2017	2017–2018	2016–2017	2017–2018
**T**_**1**_: Absolute control N:P_2_O_5_:K_2_O @ 0:0:0 kg ha^−1^	–	–	–	–	–	–	–	–
**T**_**2**_: RDF of N:P_2_O_5_:K_2_O @ 120:60:60 kg ha^−1^	9.48^c^	11.07^c^	28.59	33.21	32.73^b^	33.21^d^	13.97^c^	14.05^c^
**T**_**3**_: 75% RDF + *Trichoderma harzianum*	12.06^bc^	15.02^abc^	32.33	34.57	37.38^b^	43.39^bc^	18.06^bc^	18.99^ab^
**T**_**4**_: 75% RDF + *Pseudomonas fluorescens*	13.09^abc^	15.80^ab^	31.98	36.53	41.09^b^	43.49^bc^	19.08^ab^	19.77^ab^
**T**_**5**_: 75% RDF + *Bacillus subtilis*	11.45^bc^	14.55^bc^	33.74	37.49	34.03^b^	38.83^ cd^	17.44^bc^	18.52^b^
**T**_**6**_: 75% RDF + *T*. *harzianum* + *P*. *fluorescens*	17.44^a^	19.38^a^	32.02	34.48	54.10^a^	55.99^a^	23.44^a^	23.34^a^
**T**_**7**_: 75% RDF + *P*. *fluorescens* + *B*. *subtilis*	15.44^ab^	17.52^ab^	34.06	39.27	40.10^b^	44.57^bc^	21.44^ab^	21.49^ab^
**T**_**8**_: 75% RDF + *T*. *harzianum* + *B*. *subtilis*	13.69^abc^	16.70^ab^	30.96	34.75	36.76^b^	48.01^b^	19.68^ab^	20.67^ab^
**T**_**9**_: 75% RDF + *T*. *harzianum* + *P*. *fluorescens* + *B*. *subtilis*	10.96^bc^	13.97^bc^	32.58	35.89	33.83^b^	38.33^ cd^	16.95^bc^	17.94^bc^

Different letters indicate significant differences at *P* ≤ 0.05 among the treatments as per DMRT.

Physiological efficiency (PE_N_) reflects better accumulation and conversion of N from source to sink. The highest PE_N_ (34.06 and 39.27 kg kg^−1^) was observed with the application of 75% RDF + *P*. *fluorescens* + *B*. *subtilis* (T_7_) during both the years of study (Table 1). Low PE_N_ of crops was recorded under 75% RDF + *T*. *harzianum* + *P*. *fluorescens* (T_6_). Plants under 75% RDF + *B*. *subtilis* (T_5_) registered 4% higher PE_N_ compared to that under 75% RDF + *T*. *harzianum* + *P*. *fluorescens* + *B*. *subtilis* (T_9_). Among the single-species bio-priming agents, *B*. *subtilis* resulted in maximum PE_N_ (33.74 and 37.49 kg kg^−1^) during both years. Individual application of *T*. *harzianum* and co-application of *T*. *harzianum* and *P*. *fluorescens* showed an equivalent effect on PE_N_.

Nitrogen use efficiency in apparent recovery efficiency (ARE_N_) varied from 32.73 to 55.99% (Table 1). The highest N use efficiency of 54.10% and 55.99% in 2016–2017 and 2017–2018, respectively, was observed under 75% RDF + *T*. *harzianum* + *P*. *fluorescens* (T_6_). Among the bio-priming treatments, the lowest ARE_N_ was obtained under triple consortium treatment (T_9_). In the first year, ARE_N_ in *P*. *fluorescens* bio-primed plants was greater than other individual bio-priming agents. However, in the second year, single bio-priming of *T*. *harzianum* and *P*. *fluorescens* recorded similar N use efficiency. In addition, improved ARE_N_ was reported in lettuce plants treated with *Trichoderma*-based biostimulants^35^.

Regarding partial factor productivity (PFP_N_), the results showed similar trends to that of ARE_N_. The response ranged between 13.97 and 23.44 kg of head kg^−1^ of N applied (Table 1). Application of 75% RDF + *T*. *harzianum* + *P*. *fluorescens* (T_6_) and 75% RDF + *T*. *harzianum* + *P*. *fluorescens* + *B*. *subtilis* (T_9_) registered highest and lowest N use efficiency, respectively. About 68% increase in PFP_N_ was found in T_6_ compared to 100% RDF (T_2_). The effect of *P*. *fluorescens* on PFP_N_ was higher than other single-species bio-priming agents. The results revealed that dual consortium treatments were more competent in converting the applied N into marketable yield. Our result was consistent with the observation of Chatterjee et al.^34^.

### Phosphorus use efficiency

Agronomic efficiency (AE_P_) was maximum under 75% RDF + *T*. *harzianum* + *P*. *fluorescens* (T_6_) which registered an increment in P use efficiency from 34.89 kg of head kg^−1^ of P applied in 2016–2017 to 38.75 kg of head kg^−1^ of P applied in 2017–2018 (Table 2). Application of triple consortium was found to reduce the AE_P_ in a significant manner. Results showed the following trend: 75% RDF + *T*. *harzianum* + *P*. *fluorescens* (T_6_) > 75% RDF + *P*. *fluorescens* + *B*. *subtilis* (T_7_) > 75% RDF + *T*. *harzianum* + *B*. *subtilis* (T_8_) > 75% RDF + *P*. *fluorescens* (T_4_) > 75% RDF + *T*. *harzianum* (T_3_) > 75% RDF + *B*. *subtilis* (T_5_) > 75% RDF + *T*. *harzianum* + *P*. *fluorescens* + *B*. *subtilis* (T_9_) > 100% RDF (T_2_). Sole application of *P*. *fluorescens* along with 75% RDF (T_6_) increased the AE_P_ by 38% and 43% in the first and second year, respectively, compared with 100% RDF (T_2_).

**Table 2:** Phosphorus use efficiency of red cabbage as influenced by bio-priming and fertilisation.

Treatments	P use efficiency							
	Agronomic efficiency (kg kg^−1^)		Physiological efficiency (kg kg^−1^)		Apparent recovery efficiency (%)		Partial factor productivity (kg kg^−1^)	
	2016–2017	2017–2018	2016–2017	2017–2018	2016–2017	2017–2018	2016–2017	2017–2018
**T**_**1**_: Absolute control N:P_2_O_5_:K_2_O @ 0:0:0 kg ha^−1^	–	–	–	–	–	–	–	–
**T**_**2**_: RDF of N:P_2_O_5_:K_2_O @ 120:60:60 kg ha^−1^	18.96^c^	22.15^c^	227.25	233.68^ab^	8.18^c^	9.50^c^	27.94^c^	28.10^c^
**T**_**3**_: 75% RDF + *Trichoderma harzianum*	24.13^bc^	30.04^abc^	231.84	241.04^a^	10.68^bc^	12.46^bc^	36.11^bc^	37.97^ab^
**T**_**4**_: 75% RDF + *Pseudomonas fluorescens*	26.18^abc^	31.60^ab^	206.31	207.53^b^	12.89^ab^	15.25^ab^	38.16^ab^	39.54^ab^
**T**_**5**_: 75% RDF + *Bacillus subtilis*	22.91^bc^	29.10^bc^	201.95	241.60^a^	11.30^bc^	12.22^bc^	34.89^bc^	37.04^b^
**T**_**6**_: 75% RDF + *T*. *harzianum* + *P*. *fluorescens*	34.89^a^	38.75^a^	213.14	221.02^ab^	16.18^a^	17.45^a^	46.87^a^	46.69^a^
**T**_**7**_: 75% RDF + *P*. *fluorescens* + *B*. *subtilis*	30.89^ab^	35.05^ab^	190.59	208.97^b^	16.23^a^	16.95^a^	42.87^ab^	42.98^ab^
**T**_**8**_: 75% RDF + *T*. *harzianum* + *B*. *subtilis*	27.38^abc^	33.40^ab^	205.72	242.77^a^	13.23^ab^	13.84^ab^	39.36^ab^	41.33^ab^
**T**_**9**_: 75% RDF + *T*. *harzianum* + *P*. *fluorescens* + *B*. *subtilis*	21.93^bc^	27.94^bc^	215.89	242.77^a^	10.14^bc^	11.64^bc^	33.91^bc^	35.88^bc^

Different letters indicate significant differences at *P* ≤ 0.05 among the treatments as per DMRT.

Phosphorus use efficiency in physiological efficiency (PE_P_) varied from 227.25 to 242.77 kg of head kg^−1^ of P applied (Table 2). In the first year, application of 75% RDF + *T*. *harzianum* (T_3_) registered maximum PE_P_ (231.84 kg kg^−1^) while application of 75% RDF + *T*. *harzianum* + *B*. *subtilis* (T_8_) and 75% RDF + *T*. *harzianum* + *P*. *fluorescens* + *B*. *subtilis* (T_9_) registered maximum PE_P_ (242.77 kg kg^−1^) during the second year. Similar to PE_N_, PE_P_ lowered down with application of 75% RDF + *T*. *harzianum* + *P*. *fluorescens* (T_6_). In the second year, individual application of *P*. *fluorescens* showed lower PE_P_ than *B*. *subtilis* and *T*. *harzianum*. Yaseen and Malhi^36^ reported a similar range of PEP in wheat, and they further claimed that P decreased P use efficiency significantly.

The effect of fertilization and bio-priming on apparent recovery efficiency (ARE_P_) is presented in Table 2. The ARE_P_ was found to be maximum (16.23%) under 75% RDF + *P*. *fluorescens* + *B*. *subtilis* (T_7_) during the first year, but during the second year, maximum (17.45%) ARE_P_ was noted under 75% RDF + *T*. *harzianum* + *P*. *fluorescens* (T_6_). Among the three bio-agents, P use efficiency was in *P*. *fluorescens* > *B*. *subtilis* > *T*. *harzianum*. The present study results revealed that bio-priming with P. fluorescens demonstrated the highest increment (18%) in AREP compared to the first year. Application of triple consortium and single bio-priming with *T*. *harzianum* resulted in a similar ARE_P_. Increased P use efficiency due to co-inoculation of rhizospheric bacterial (endophytic) agents was reported by Emami et al.^37^.

Partial factor productivity (PFP_P_) ranged from 27.94 to 46.87 kg of head kg^−1^ of P applied with an average value of 37.51 kg of head kg^−1^ of P applied during the first year and 38.69 kg of head kg^−1^ of P applied during the second year (Table 2). Among the bio-priming treatments, the application of 75% RDF + *T*. *harzianum* + *P*. *fluorescens* (T_6_) and 75% RDF + *T*. *harzianum* + *P*. *fluorescens* + *B*. *subtilis* (T_9_) recorded the highest and lowest PFP_P_, respectively. Compared with 100% RDF (T_2_), T_6_ increased the P use efficiency by 67%. *P. fluorescens* exhibited the highest P use efficiency regarding individual bio-priming agents, followed by *T*. *harzianum* and *B*. *subtilis*. However, compared with the first year, bio-priming with *B*. *subtilis* showed a 6% increment in PFP_P_ during the second year. The results of PFP_P_ followed the order: dual-species bio-priming > single-species bio-priming > triple-species bio-priming.

### Potassium use efficiency

The results of K use efficiency in the form of agronomic efficiency (AE_K_), and partial factor productivity (PFP_K_) was similar to that of P use efficiency (AE_P_ and PFP_P_) because the amount of nutrient applied for P and K was the same, i.e., 60 kg as RDF. The maximum and minimum K use efficiency (AE_K_ and PFP_K_) were obtained from 75% RDF + *T*. *harzianum* + *P*. *fluorescens* (T_6_) and 100% RDF (T_2_), respectively (Table 3). The performance of the triple consortium was lowest among the bio-primed treatments. Application of *P*. *fluorescens* resulted in higher AE_K_ in terms of single-species bio-priming.

**Table 3:** Potassium use efficiency of red cabbage as influenced by bio-priming and fertilisation.

Treatments	K use efficiency							
	Agronomic efficiency (kg kg^−1^)		Physiological efficiency (kg kg^−1^)		Apparent recovery efficiency (%)		Partial factor productivity (kg kg^−1^)	
	2016–201,717	2017–2018	2016–2017	2017–2018	2016–2017	2017–2018	2016–2017	2017–2018
**T**_**1**_: Absolute control N:P_2_O_5_:K_2_O @ 0:0:0 kg ha^−1^	–	–	–	–	–	–	–	–
**T**_**2**_: RDF of N:P_2_O_5_:K_2_O @ 120:60:60 kg ha^−1^	18.96^c^	22.15^c^	32.08	34.33	59.13^d^	64.27^c^	27.94^c^	28.10^c^
**T**_**3**_: 75% RDF + *Trichoderma harzianum*	24.13^bc^	30.04^abc^	32.31	33.93	74.45^ cd^	88.44^abc^	36.11^bc^	37.97^ab^
**T**_**4**_: 75% RDF + *Pseudomonas fluorescens*	26.18^abc^	31.60^ab^	31.25	33.49	84.02^abcd^	94.83^ab^	38.16^ab^	39.54^ab^
**T**_**5**_: 75% RDF + *Bacillus subtilis*	22.91^bc^	29.10^bc^	29.47	33.29	77.54^bcd^	87.75^abc^	34.89^bc^	37.04^b^
**T**_**6**_: 75% RDF + *T*. *harzianum* + *P*. *fluorescens*	34.89^a^	38.75^a^	31.68	33.67	109.26^a^	116.49^a^	46.87^a^	46.69^a^
**T**_**7**_: 75% RDF + *P*. *fluorescens* + *B*. *subtilis*	30.89^ab^	35.05^ab^	29.18	31.32	105.92^ab^	112.53^ab^	42.87^ab^	42.98^ab^
**T**_**8**_: 75% RDF + *T*. *harzianum* + *B*. *subtilis*	27.38^abc^	33.40^ab^	29.35	32.09	93.83^abc^	104.45^ab^	39.36^ab^	41.33^ab^
**T**_**9**_: 75% RDF + *T*. *harzianum* + *P*. *fluorescens* + *B*. *subtilis*	21.93^bc^	27.94^bc^	30.60	32.69	71.51^ cd^	84.97^bc^	33.91^bc^	35.88^bc^

Different letters indicate significant differences at *P* ≤ 0.05 among the treatments as per DMRT.

Physiological efficiency (PE_K_) varied from 29.18 to 34.33 kg of head kg^−1^ of K applied (Table 3). The highest PE_K_ of 32.31 kg of head kg^−1^ of K applied was recorded with 75% RDF + *T*. *harzianum* (T_3_) in 2016–2017, while application of 100% RDF (T_2_) registered the highest PE_K_ of 34.33 kg of head kg^−1^ of K applied in 2017–2018. Results of PE_K_ further indicated that microbial consortium treatments gave an equivalent effect to that of single-species bio-priming treatments. Sole application of *T*. *harzianum* resulted in higher PE_K_ than other bio-primed treatments including dual and triple consortiums.

In the present study, apparent recovery efficiency (ARE_K_) ranged from 59.13 to 116.49%, with a mean value of 89.34% (Table 3). The maximum ARE_K_ was achieved under 75% RDF + *T*. *harzianum* + *P*. *fluorescens* (T_6_), which increased from 109.26% in the first year to 116.49% in the second year. Among single bio-priming agents, *P*. *fluorescens* showed the highest ARE_K_. This microbe recorded a 13% increment in ARE_K_ as compared to the first year. Results followed the trend of 75% RDF + dual consortium > 75% RDF + single bio-priming agents > 75% RDF + triple consortium > 100% RDF. Application of triple consortium reduced the ARE_K_ by 45% (average of two years) compared to that of dual consortium (*T*. *harzianum* + *P*. *fluorescens*). Enhanced K uses efficiency in AE_K_, PE_K_, and ARE_K_ due to bacterial inoculations and reduced chemical fertilizer quantity reported by Khanghah et al.^38^.

### Yield attributes

**Figure 2:**
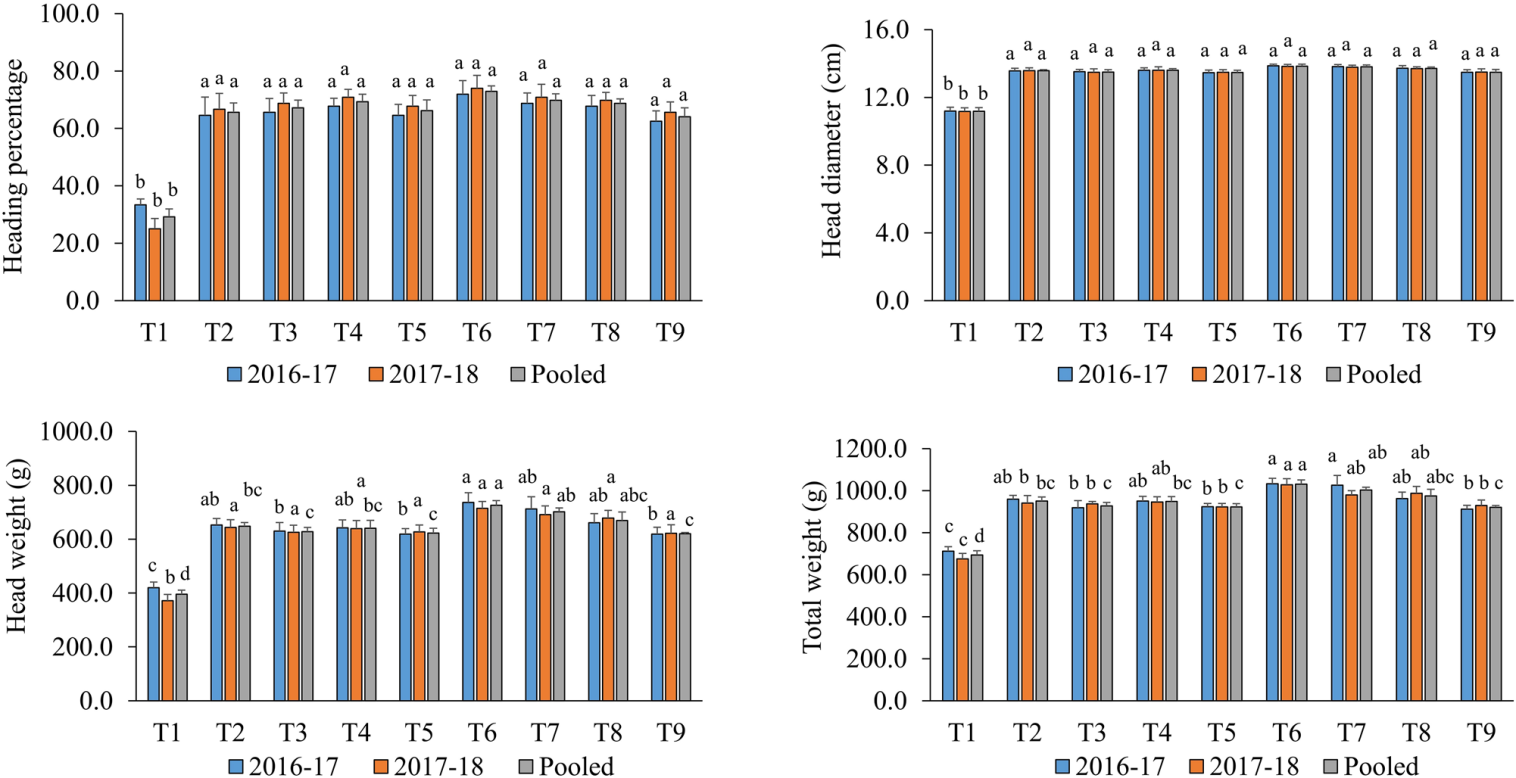
Effect of bio-priming and fertilization on yield attributes of red cabbage. Bars (mean ± SE; *n* = 3) followed by different alphabets significantly differ (*P* ≤ 0.05) among the treatments as per DMRT. Treatments: T_1_: Absolute control N:P_2_O_5_:K_2_O @ 0:0:0 kg ha^−1^; T_2_: RDF of N:P_2_O_5_:K_2_O @ 120:60:60 kg ha^−1^; T_3_: 75% RDF + *Trichoderma harzianum*; T_4_: 75% RDF + *Pseudomonas fluorescens*; T_5_: 75% RDF + *Bacillus subtilis*; T_6_: 75% RDF + *T*. *harzianum* + *P*. *fluorescens*; T_7_: 75% RDF + *P*. *fluorescens* + *B*. *subtilis*; T_8_: 75% RDF + *T*. *harzianum* + *B*. *subtilis*; T_9_: 75% RDF + *T*. *harzianum* + *P*. *fluorescens* + *B*. *subtilis.*

The observation on heading percentage and head diameter indicated that the sole application of mineral fertilizers and integrated application along with bio-agents did not bring any significant variation among the treated plants (Fig. 2). Heading percentage (72.92), as well as head diameter (13.85), was recorded to be maximum in 75% RDF + *T*. *harzianum* + *P*. *fluorescens* (T_6_). The lowest heading percentage was noticed in absolute control (29.17) followed by a triple consortium (64.06). The average diameter of marketable heads was 13.35 cm. Head weight varied from 371.63 to 736.29 g (Fig. 2).

On a pooled basis, a significantly higher head weight (725.51 g) was detected with T_6_ compared to the rest of the treatments. It registered 12% and 83% increments over 100% RDF (T_2_) and absolute control (T_1_), respectively. Bio-priming with triple consortium recorded 17% lower head weight than dual consortium (*T*. *harzianum* + *P*. *fluorescens*). Results on head weight followed the order of 75% RDF + *T*. *harzianum* + *P*. *fluorescens* (T_6_) > 75% RDF + *P*. *fluorescens* + *B*. *subtilis* (T_7_) > 75% RDF + *T*. *harzianum* + *B*. *subtilis* (T_8_) > 100% RDF (T_2_) > 75% RDF + *P*. *fluorescens* (T_4_) > 75% RDF + *T*. *harzianum* (T_3_) > 75% RDF + *B*. *subtilis* (T_5_) > 75% RDF + *T*. *harzianum* + *P*. *fluorescens* + *B*. *subtilis* (T_9_) > absolute control (T_1_). A perusal of pooled data presented in Fig. 2 revealed that the total weight varied from 693.33 to 1030.49 g. In total weight, T_6_ was 8% and 49% higher than T_2_ and T_1_, respectively. The total weight of plants under T_6_ was at par with T_7_. *P*. *fluorescens* combined with 75% RDF resulted in an equivalent total weight to that of 100% RDF. The direct impact of bio-priming on plant growth promotion was reported in earlier studies^5,8^. However, these studies were conducted in pot conditions. The current study focuses on the practical utility of such technology under field conditions. Higher yield attributes in bio-primed treatments could alter cellular mechanisms in these plants and regulated nutrient supply from the soil. Integrated application of inorganic fertilizers (75% RDF) and organics (biofertilizer + vermicompost) yielded the highest marketable head percentage and a head weight of cabbage^39^. Application of organics (poultry manure) alone showed greater head weight (17%) and head length (8%) of cabbage over NPK fertilizers^40^.

### Energy budgeting

Efficient use of energy inputs in a production system must lessen our dependence on non-renewable energy and maintain sustainability. The magnitude of energy input ranged from 6323 to 14,663 MJ ha^−1^ (Table 4). Bio-priming intervention in red cabbage cultivation saved 1906 to 1965 MJ ha^−1^ energy requirement. As expected, the energy consumed under 100% RDF (T_2_) was ~ 15% higher than bio-priming treatments. Energy output (26,370 and 26,630 MJ ha^−1^) and energy balance (13,643 and 13,903 MJ ha^−1^) were considerably higher in T_6_ treatment (75% RDF + *T*. *harzianum* + *P*. *fluorescens*) during both the years of study. Compared to the first year, energy balance declined (2%) in T_2_ treatment. Among the treatments with amendments, T_6_ showed the highest energy use efficiency (2.09). The triple consortium treatment (T_6_) recorded the lowest energy balance (9799 MJ ha^−1^) and energy use efficiency (1.77) compared to other bio-priming treatments. However, the bio-priming treatments were greater in energy balance and energy use efficiency than sole use of chemical fertilization. It shows that fertilizer inputs consume the highest energy. In cabbage production, the consumption of energy by mineral fertilizers may be as high as 77%^20^. It is of great concern, and the non-renewable sources of plant nutrients must be substituted with renewable sources. Mihov et al.^41^ reported that cabbage's organic production could save 31.23% energy unit area^−1^ than its conventional production system. Application of biofertilizers and reduced dose of fertilizers enhance energy balance and energy use efficiency of a cropping system^42–48^.

**Table 4:** Energetics of red cabbage production as influenced by bio-priming and mineral fertilization.

Treatments	2016–2017				2017–2018			
	Input energy (MJ ha^−1^)	Output energy (MJ ha^−1^)	Energy balance (MJ ha^−1^)	Energy use efficiency	Input energy (MJ ha^−1^)	Output energy (MJ ha^−1^)	Energy balance (MJ ha^−1^)	Energy use efficiency
**T**_**1**_: Absolute control N:P_2_O_5_:K_2_O @ 0:0:0 kg ha^−1^	6323	13553^d^	7230^d^	2.14^a^	6323	12187^c^	5863^d^	1.93^ab^
**T**_**2**_: RDF of N:P_2_O_5_:K_2_O @ 120:60:60 kg ha^−1^	14,663	23137^abc^	8473^ cd^	1.58^e^	14,663	22967^b^	8303^ cd^	1.57^c^
**T**_**3**_: 75% RDF + *Trichoderma harzianum*	12,698	22307^bc^	9609^bcd^	1.76^cde^	12,698	23430^b^	10732^bc^	1.85^ab^
**T**_**4**_: 75% RDF + *Pseudomonas fluorescens*	12,698	23477^abc^	10779^abc^	1.85^bcd^	12,698	23947^ab^	11249^abc^	1.89^ab^
**T**_**5**_: 75% RDF + *Bacillus subtilis*	12,698	22207^bc^	9509^bcd^	1.75^cde^	12,698	22790^b^	10092^bc^	1.79^bc^
**T**_**6**_: 75% RDF + *T*. *harzianum* + *P*. *fluorescens*	12,727	26370^a^	13643^a^	2.07^ab^	12,727	26630^a^	13903^a^	2.09^a^
**T**_**7**_: 75% RDF + *P*. *fluorescens* + *B*. *subtilis*	12,727	25303^ab^	12576^ab^	1.99^abc^	12,727	24867^ab^	12139^ab^	1.95^ab^
**T**_**8**_: 75% RDF + *T*. *harzianum* + *B*. *subtilis*	12,727	23770^abc^	11043^abc^	1.87^bcd^	12,727	24657^ab^	11929^ab^	1.94^ab^
**T**_**9**_: 75% RDF + *T*. *harzianum* + *P*. *fluorescens* + *B*. *subtilis*	12,757	21640^c^	8883^ cd^	1.70^de^	12,757	22557^b^	9799^bc^	1.77^bc^

Different letters indicate significant differences at *P* ≤ 0.05 among the treatments as per DMRT.

### Economic analysis

Farmers will adopt any technology when it is economically feasible. Different economic indicators, such as gross return, the net return, and benefit:cost (B:C) ratio showed wide variations among the treatments (Table 5). Maximum gross return (US $ 16,030 and 15,966 ha^−1^) and net return (US $ 13,877 and 13,813 ha^−1^) were recorded with the application of T_6_ treatment (75% RDF + *T*. *harzianum* + *P*. *fluorescens*) during both the years of study. The treatment also achieved the highest B:C ratio of 6.45 and 6.42. Lowest returns and B:C ratio were observed in control. Our results showed that the application of microbial consortium and 75% RDF could be profitable than 100% RDF by providing about US $ 3222.5 higher net return on a hectare basis. The integrated approach's performance was better in a dual consortium in most cases, while in single-species bio-priming, application of *P*. *fluorescence* yields a higher B:C ratio than 100% RDF. The high profitability of a system is related to higher productivity and lowering of production cost. Thakur et al.^49^ noted the highest net return and B:C ratio with the conjoint application of 75% NPK and organics (organic manures + biofertilizers) in cauliflower. Another study by Kamal et al.^50^ reported a significant effect on B:C ratio of hybrid cabbage production due to the combined application of bio-agents and chemical fertilizers.

**Table 5:** Economics of red cabbage production as influenced by bio-priming and mineral fertilization.

Treatments	2016–2017			2017–2018		
	Gross return (US $ ha^−1^)	Net return (US $ ha^−1^)	B:C ratio	Gross return (US $ ha^−1^)	Net return (US $ ha^−1^)	B:C ratio
**T**_**1**_: Absolute control N:P_2_O_5_:K_2_O @ 0:0:0 kg ha^−1^	4098^c^	2011^c^	0.96^c^	2714^c^	627^c^	0.30^c^
**T**_**2**_: RDF of N:P_2_O_5_:K_2_O @ 120:60:60 kg ha^−1^	12743^ab^	10588^ab^	4.91^ab^	12812^b^	10657^b^	4.95^b^
**T**_**3**_: 75% RDF + *Trichoderma harzianum*	12350^b^	10200^b^	4.74^b^	12987^b^	10836^b^	5.04^b^
**T**_**4**_: 75% RDF + *Pseudomonas fluorescens*	13050^ab^	10900^ab^	5.07^ab^	13522^ab^	11372^ab^	5.29^ab^
**T**_**5**_: 75% RDF + *Bacillus subtilis*	11932^b^	9782^b^	4.55^b^	12667^b^	10517^b^	4.89^b^
**T**_**6**_: 75% RDF + *T*. *harzianum* + *P*. *fluorescens*	16030^a^	13877^a^	6.45^a^	15966^a^	13813^a^	6.42^a^
**T**_**7**_: 75% RDF + *P*. *fluorescens* + *B*. *subtilis*	14662^ab^	12509^ab^	5.81^ab^	14700^ab^	12547^ab^	5.83^ab^
**T**_**8**_: 75% RDF + *T*. *harzianum* + *B*. *subtilis*	13462^ab^	11308^ab^	5.25^ab^	14136^ab^	11983^ab^	5.57^ab^
**T**_**9**_: 75% RDF + *T*. *harzianum* + *P*. *fluorescens* + *B*. *subtilis*	11596^b^	9440^b^	4.38^b^	12271^b^	10115^b^	4.69^b^

1 US $ = 66 INR; Produce cost calculated @ 0.76 US $ kg^−1^; Spencer (Supermarket) cost = 2.42 US $ kg^−1^.

Different letters indicate significant differences at *P* ≤ 0.05 among the treatments as per DMRT.

## Conclusion

We have evaluated different performance indicators, each depicting new viewpoints on biotechnological intervention in integrated nutrient management. Our results generated from field experiments indicated bio-priming in combination with mineral fertilization augmented the productivity, nutrient use efficiency, and profitability of red cabbage cultivation while minimizing the energy requirements. The strategy was more effective under dual inoculation treatments over control. Bio-priming with *T*. *harzianum* and *P*. *fluorescens* emerged out to be the most suitable treatment over control for red cabbage production, followed by bacterial co-inoculations of *P*. *fluorescens* and *B*. *subtilis*. Development of low-cost technology with a high B:C ratio has prime importance for resource-poor farmers. Our investigation also has great relevance to the United Nations Sustainable Development Goals' targets by adopting cleaner and cost-effective crop production practices to ensure food security and resource use efficiency in developing countries like India.

## Materials and methods

### Study site

**Figure 3:**
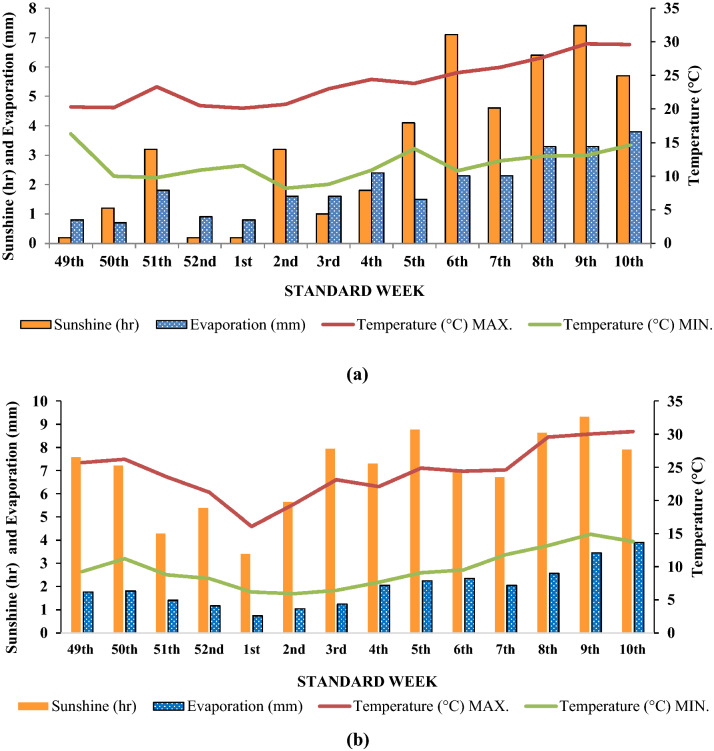
Meteorological observations during growth period of red cabbage (**a**) 2016–2017 (**b**) 2017–208.

A two-year field experiment was conducted at the Vegetable Research Farm of the Institute of Agricultural Sciences, Banaras Hindu University, Varanasi, (25^o^26’N, 82^o^99’E, and 80.7 m above mean sea level), Uttar Pradesh, India, during two consecutive winter (*rabi*) seasons of 2016–17 and 2017–18. The experimental site falls under Middle Gangetic Plains (agro-ecological region) of Eastern India. Varanasi is characterized by a semi-arid and sub-humid climate with an annual average rainfall of around 1100 mm. The cold period starts in November and stays till February, designating January as the coldest month. Meteorological observations (maximum and minimum temperature, sunshine, and evaporation) during the cropping seasons are graphically presented in Fig. 3. The mean weekly maximum and minimum temperature of the two crop seasons were recorded as 30.0 °C and 7.06 °C, respectively. Sunshine duration was higher in the second growing season. The experimental soil, Gangetic alluvial in nature, is classified in Typic Ustochrept of the order Inceptisol. Salient initial physical, chemical and biological properties (Table 6) of the soil were determined before starting field experiments. The initial analysis revealed that the soil was sandy loam in texture, slightly alkaline in reaction, low in organic carbon and available N content, and medium in available P and available K content.

**Table 6:** Initial properties of the soil before transplanting of crop.

Parameter	Value		Reference
	2016–2017	2017–2018	
Sand (%)	47.65	47.63	^51^
Silt (%)	30.06	30.12	
Clay (%)	22.29	22.25	
Bulk density (Mg m^−3^)	1.36 ± 0.01	1.37 ± 0.01	^52^
Maximum water holding capacity (%)	42.4	42.1	^53^
pH (1:2)	7.58 ± 0.02	7.52 ± 0.03	^54^
EC (dS m^−1^)	0.25 ± 0.01	0.26 ± 0.01	^55^
Exchangeable Ca^2+^ + Mg^2+^ [cmol (p +) kg^−1^]	8.72 ± 0.29	9.16 ± 0.23	^56^
CEC [cmol (p +) kg^−1^]	18.74 ± 0.39	19.86 ± 0.53	^57^
Organic C (g kg^−1^)	4.04 ± 0.16	4.15 ± 0.20	^58^
Available N (kg ha^−1^)	203.21 ± 9.22	213.25 ± 8.87	^59^
Available P (kg ha^−1^)	20.86 ± 0.44	22.35 ± 0.31	^60^
Available K (kg ha^−1^)	217.73 ± 2.42	226.69 ± 6.40	^61^

Values are ± standard error of mean.

### Seed and microbial agents

Red cabbage variety F1 Hybrid Red Ruby-2 was used as a test crop in the present study. The maturity period of the crop lies between 90 and 100 days after transplanting. Truthful label seeds with 70% germination rate and 98% physical purity were used for sowing in the nursery. The variety is suitable for growing in the Eastern Zone of India. Microbes, viz., *Trichoderma harzianum* (BHU P4; GenBank accession No. MH730446), *Pseudomonas fluorescens* (OKC; GenBank accession No. JN128891), and *Bacillus subtilis* (BHHU100; GenBank accession No. JN099686) were collected from the Department of Mycology and Plant Pathology of Institute of Agricultural Sciences, Banaras Hindu University, India. These microbial strains were compatible with each other and showed particular plant growth-promoting (PGP) traits^62^.

### Bio-priming

#### Preparation of inoculum

Bacterial (*B*. *subtilis* and *P*. *fluorescens*) culture was inoculated in 250 mL flasks containing 100 mL nutrient broth and kept in a shaking incubator (150 rpm; 28 ± 2 °C) for 48 h. Bacterial pellets were obtained by centrifugation (7000 rpm; 4 °C) for 10 min. Discarding the supernatant, the cell pellets were soon washed with sterile distilled water. An adjustment of final cell density to 4 × 10^8^ CFU mL^−1^ was done using optical density (< 1) at 600 nm^63^. In *T*. *harzianum*, spore suspension was prepared from 1 week of culture grown (28 ± 2 °C) on potato dextrose agar by harvesting the spores in sterilized 0.85% sodium chloride (NaCl)^5^. The spore concentration was adjusted to 2 × 10^7^ CFU mL^−1^ by measuring the optical density in a spectrophotometer. Cell suspensions and/or spore suspension were mixed in equal ratios for the dual consortium (1:1) and triple consortium (1:1:1).

#### Seedling bio-priming

Red cabbage seeds were washed with tap water and surface sterilized with 0.1% mercuric chloride (HgCl_2_) solution for 2 min. After sterilization, the seeds were immediately washed with autoclaved distilled water three times. Next, the seeds were soaked in sterile distilled water for 2 h. After the hydration treatment, the seeds were sown in the nursery. Seedlings were picked when there were 5–6 leaves. Soil attached with the roots was washed carefully, followed by root dipping in liquid culture containing 2% carboxymethyl cellulose (CMC) as an adhesive agent. The bio-priming process was followed for 5 h under incubated conditions (28 ± 2 °C; > 90% relative humidity)^5,36,62^.

#### Experimental design and crop management

The field setup was laid out in a randomized block design with nine treatments and three replications. Red cabbage seeds were sown in raised bed nursery about 1 month before transplanting. The field was twice ploughed by a tractor and planked 15 days before the implementation of the experiment. Crop residues, stones, pebbles, or weeds were removed manually from the field. Five weeks of healthy and uniform-sized red cabbage seedlings were transplanted in 4 × 2 m^2^ plots with a spacing of 50 cm × 50 cm on 5^th^ December 2016 and 2017. Four rows in a plot accommodated 32 plants. The crop was irrigated for 2 weeks with a watering can just after transplanting. After that, it received four irrigations at 7–10 days intervals as per the requirement. A full dose of diammonium phosphate (DAP) and muriate of potash (MOP) was applied as basal at the time of final land preparation. The urea dose was given in three splits, including basal application and top dressing, at 30 and 45 days (head initiation) after transplanting plant seedlings. Other intercultural operations like gap filling and weeding (3 times) were also carried. In general, the pest and disease incidence was not observed during both crop seasons. The treatments comprised nine combinations of mineral fertilizers and bio-agents, including one absolute control outlined in Table 7. The recommended dose of fertilizer (RDF) was applied @ 120:60:60 kg ha^−1^ (N:P_2_O_5_:K_2_O) through urea, DAP, and MOP, respectively. The fertilizer dose was reduced to 25% when the priming agents were used (Table 7).

**Table 7:** Details of treatment combinations applied during the study.

Treatments	Notations used	Mineral fertilization (kg ha^−1^)		
		N	P_2_O_5_	K_2_O
Absolute control N:P_2_O_5_:K_2_O @ 0:0:0 kg ha^−1^	T_1_	0	0	0
RDF of N:P_2_O_5_:K_2_O @ 120:60:60 kg ha^−1^	T_2_	120	60	60
75% RDF + *Trichoderma harzianum*	T_3_	90	45	45
75% RDF + *Pseudomonas fluorescens*	T_4_	90	45	45
75% RDF + *Bacillus subtilis*	T_5_	90	45	45
75% RDF + *T*. *harzianum* + *P*. *fluorescens*	T_6_	90	45	45
75% RDF + *P. fluorescens* + *B*. *subtilis*	T_7_	90	45	45
75% RDF + *T*. *harzianum* + *B*. *subtilis*	T_8_	90	45	45
75% RDF + *T*. *harzianum* + *P*. *fluorescens* + *B*. *subtilis*	T_9_	90	45	45

#### Biometric observations

Data related to yield attributing parameters such as heading percentage, head diameter (cm), head weight (g), and total weight (g) were collected at harvest.

#### Computation of nutrient uptake and nutrient use efficiency

Nutrient uptake and nutrient use efficiency were calculated using the following equations.

#### Nutrient uptake

$${\text{Nutrient }}\;{\text{uptake }}({\text{kg }}\;{\text{ha}}^{ - 1} ) = \frac{{{\text{Nutrient }}\;{\text{content }}\left( \% \right) \times {\text{Yield}} ({\text{kg }}\;{\text{ha}}^{ - 1} ) }}{100}$$

Nitrogen content was determined by the micro Kjeldahl method after pre-digestion with concentrated sulphuric acid (H_2_SO_4_) followed by catalyst mixture^64^. Phosphorus content was determined by vanadomolybdophosphoric yellow color method after pre-digestion with diacid (HNO_3_:HClO_4_)^65^. The acid-digest prepared for P was used to assess potassium (K) in a flame photometer^66^.

#### Agronomic efficiency

$${\text{Agronomic }}\;{\text{efficiency }}\left( {{\text{kg}}/{\text{ha}}} \right) = \frac{{{\text{Y}} - {\text{Y}}_{{\text{o}}} }}{{{\text{Quantity}} \left( {{\text{kg}}/{\text{ha}}} \right)\;{\text{ of}}\;{\text{ nutrients}}\;{\text{ applied}}\; 100}}$$

where Y = head yield (kg ha^−1^) with applied nutrient, Y_0_ = head yield (kg ha^−1^) with no applied nutrient.

#### Physiological efficiency

$${\text{Physiological }}\;{\text{efficiency }}\;\left( {{\text{kg}}\;{\text{ kg}}^{ - 1} } \right) = \frac{{{\text{Y}} - {\text{Y}}_{{\text{O}}} }}{{{\text{U}} - {\text{U}}_{{\text{O}}} }}$$

where, Y = head yield (kg ha^−1^) with applied nutrient, Y_0_ = head yield (kg ha^−1^) with no applied nutrient, U = total nutrient uptake (kg ha^−1^) with applied nutrient; U_0_ = total nutrient uptake (kg ha^−1^) with no applied nutrient.

#### Apparent recovery efficiency

$${\text{Apparent}}\;{\text{recovery}}\;{\text{efficiency }}\left( \% \right) = \frac{{{\text{U}} - {\text{U}}_{O} }}{{{\text{Quantity}} \left( {{\text{kg}}\;{\text{ ha}}^{ - 1} } \right) \;{\text{of}}\;{\text{ nutrient}}\; {\text{applied}}}} \times 100$$

where, U = total nutrient uptake (kg ha^−1^) with applied nutrient; U_0_ = total nutrient uptake (kg ha^−1^) with no applied nutrient.

##### Partial factor productivity

$${\text{Partial}}\;{\text{ factor}}\;{\text{ productivity}} \left( {{\text{kg}}\;{\text{ kg}}^{ - 1} } \right) = \frac{{{\text{Head}}\;{\text{ yield}} \left( {{\text{kg }}\;{\text{ha}}^{ - 1} } \right) }}{{{\text{Quantity}}\; \left( {{\text{kg}}\;{\text{ha}}^{ - 1} } \right) \;{\text{of}}\;{\text{ nutrient}}\; {\text{applied}}}}$$

##### Energy analysis

The energy input–output relationship was determined based on the energy equivalent of inputs and output (Table 8). Solar energy was not included in the calculation. Energy analysis involved the following equations.

$${\text{Energy}}\;{\text{ use}}\;{\text{ efficiency}} = \frac{{{\text{Energy}}\;{\text{ output}} \left( {{\text{MJ }}\;{\text{ha}}^{ - 1} } \right)}}{{{\text{Energy }}\;{\text{input}} \left( {{\text{MJ}}\;{\text{ha}}^{ - 1} } \right)}}$$

$${\text{Energy}}\;{\text{ productivity}} = \frac{{{\text{Head }}\;{\text{yield }}\left( {{\text{kg}}\; {\text{ha}}^{ - 1} } \right)}}{{{\text{Energy }}\;{\text{input}} \left( {{\text{MJ}}\;{\text{ha}}^{ - 1} } \right)}}$$

$${\text{Energy}}\;{\text{ balance}} = {\text{Energy }}\;{\text{output }}\;\left( {{\text{MJ}}\;{\text{ ha}}^{ - 1} } \right) - {\text{Energy}}\;{\text{input}} \left( {{\text{MJ }}\;{\text{a}}^{ - 1} } \right)$$

**Table 8:** Energy equivalents and cost of inputs and outputs in red cabbage production.

Particulars	Units	Energy equivalent (MJ unit^−1^ ^20,21,41^)	Unit cost (US $ unit^−1^)
Human labor	hr	1.96	1.26
Diesel	L	56.31	0.98
Farm machinery	hr	62.70	7.57
Electricity	kWh	11.91	
Mineral fertilizer			
N	kg	60.60	0.227
P_2_O_5_	kg	11.10	0.378
K_2_O	kg	6.70	0.303
Biofertilizer	L	2.98	7.57
Irrigation water	m^3^	1.02	0.0076
Seed	kg	0.8	454
Produce	kg	0.8	0.76

1 US $ = 66 INR.

### Economic analysis

Expenditures were calculated considering the unit cost of variable inputs and output (produce) based on prevailing market prices (Table 8). The cost of cultivation (total expenditure) was subtracted from the gross return for obtaining the net return. Benefit: cost (B:C) ratio was worked out using the following formula.

$${\mathrm{B:C ratio}} = \frac{{{\mathrm{Net}}\;{\mathrm{ return}} \left( {{\mathrm{US }}\;{{\$ }}\,{\mathrm{ ha}}^{ - 1} } \right)}}{{\text{Total}\, exp \text{enditure} \left( {{\mathrm{US}}\;\$ \,{\mathrm{ha}}^{ - 1} } \right)}}$$

### Statistical analysis

Data collected during the study were subjected to one-way analysis of variance (ANOVA). Values are presented as mean ± standard error (SE). Significance of difference between treatment means was performed using Duncan’s multiple range test (DMRT) at *P* ≤ 0.05 significance level. Statistical Package for Social Science (SPSS, version 20) software was used for these analyses ^67,68^.

### International, national and/or institutional guidelines

Authors reporting experiments confirmed that the use of plants in the present study complies with international, national and/or institutional guidelines.

## Supplementary Information

Supplementary Information.
