# Supplementary material for: Optimizing nutrient use efficiency, productivity, energetics, and economics of red cabbage following mineral fertilization and biopriming with compatible rhizosphere microbes
Source: Sci Rep. 2021 Aug 3;11:15680. doi: 10.1038/s41598-021-95092-6 (PMC8333308; doi:10.1038/s41598-021-95092-6)
Supplement: Supplementary file 1 — Supplementary Information. [file 41598_2021_95092_MOESM1_ESM.docx]

**Optimizing nutrient use efficiency, productivity, energetics, and economics of red cabbage following mineral fertilization and biopriming with compatible rhizosphere microbes**

Deepranjan Sarkar^1^, Ardith Sankar^2^, O. Siva Devika^1^, Sonam Singh^1^, Shikha^3^, Manoj Parihar^4^, Amitava Rakshit^1^*, R. Z. Sayyed^5^, Abdul Gafur^6^, Mohammad Javed Ansari^7^, Subhan Danish^8^*, Shah Fahad^9^, Rahul Datta^10^

^1^Department of Soil Science and Agricultural Chemistry, Institute of Agricultural Sciences, Banaras Hindu University, Varanasi-221005, Uttar Pradesh, India; [deep.gogreen@gmail.com](mailto:deep.gogreen@gmail.com); [shivadevika990@gmail.com](mailto:shivadevika990@gmail.com); [sonamsingh791@yahoo.com](mailto:sonamsingh791@yahoo.com); [amitavar@bhu.ac.in](mailto:amitavar@bhu.ac.in)

^2^Department of Agronomy, Institute of Agricultural Sciences, Banaras Hindu University, Varanasi-221005, Uttar Pradesh, India; [ardithsankar777@gmail.com](mailto:ardithsankar777@gmail.com)

^3^Krishi Vigyan Kendra, Ranichauri, Veer Chandra Singh Garhwali Uttarakhand University of Horticulture and Forestry, Tehri Garhwal-249199, Uttarakhand, India; [shikha9104@gmail.com](mailto:shikha9104@gmail.com)

^4^Crop Production Division, ICAR-Vivekananda Parvatiya Krishi Anusandhan Sansthan, Almora, India; [manojbhu7@gmail.com](mailto:manojbhu7@gmail.com)

^5^Department of Microbiology, PSGVP Mandal’s, Arts, Science & Commerce College, Shahada 425409, Maharashtra, India

^6^Sinarmas Forestry Corporate Research and Development, Perawang 28772, Indonesia; gafur@uwalumni.com

^7^Department of Botany, Hindu College Moradabad (Mahatma Jyotiba Phule Rohilkhand Univesity Bareilly), Uttar Pradesh 244001, India; mjavedansari@gmail.com

^8^Department of Soil Science, Faculty of Agricultural Sciences and Technology, Bahauddin Zakariya University, Multan, 60800 Pakistan; [sd96850@gmail.com](mailto:sd96850@gmail.com)

^9^Department of Agronomy, The University of Haripur, Haripur 22620, Pakistan; [shah_fahad80@yahoo.com](mailto:shah_fahad80@yahoo.com)

^10^Department of Geology and Pedology, Faculty of Forestry and Wood Technology, Mendel University in Brno, Zemedelska1, 61300 Brno, Czech Republic; [rahulmedcure@gmail.com](mailto:rahulmedcure@gmail.com)

*Corresponding author E-mail: [sd96850@gmail.com](mailto:sd96850@gmail.com); [amitavar@bhu.ac.in](mailto:amitavar@bhu.ac.in)

**Supplementary tables**

**Table S1.** Effect of bio-priming and fertilisation on N uptake by red cabbage

| **Treatments** | **N uptake (kg ha^-1^)** | | | | | |
| --- | --- | --- | --- | --- | --- | --- |
|  | **2016-17** | | | **2017-18** | | |
|  | **Head** | **Stalk** | **Total** | **Head** | **Stalk** | **Total** |
| **T_1_**: Absolute control N:P_2_O_5_:K_2_O @ 0:0:0 kg ha^-1^ | 7.09 ± 0.44^d^ | 14.43 ± 0.39^b^ | 21.53 ± 0.83^c^ | 4.55 ± 0.94^c^ | 15.02 ± 0.05^b^ | 19.56 ± 0.92^d^ |
| **T_2_**: RDF of N:P_2_O_5_:K_2_O @ 120:60:60 kg ha^-1^ | 36.41 ± 5.29^bc^ | 24.39 ± 1.63^a^ | 60.80 ± 5.82^ab^ | 37.51 ± 3.03^b^ | 21.91 ± 1.26^a^ | 59.41 ± 1.95^bc^ |
| **T_3_**: 75% RDF + *Trichoderma harzianum* | 31.92 ± 2.39^bc^ | 23.25 ± 1.22^a^ | 55.17 ± 2.87^b^ | 35.06 ± 4.05^b^ | 23.56 ± 2.34^a^ | 58.62 ± 1.79^bc^ |
| **T_4_**: 75% RDF + *Pseudomonas fluorescens* | 34.55 ± 2.77^bc^ | 23.96 ± 0.93^a^ | 58.51 ± 3.48^ab^ | 35.16 ± 0.26^b^ | 23.54 ± 1.99^a^ | 58.70 ± 1.99^bc^ |
| **T_5_**: 75% RDF + *Bacillus subtilis* | 28.86 ± 1.59^c^ | 23.29 ± 1.69^a^ | 52.15 ± 3.25^b^ | 31.90 ± 0.87^b^ | 22.61 ± 1.03^a^ | 54.51 ± 1.12^c^ |
| **T_6_**: 75% RDF + *T*. *harzianum* + *P*. *fluorescens* | 46.96 ± 4.76^a^ | 23.25 ± 2.51^a^ | 70.21 ± 5.48^a^ | 46.38 ± 3.63^a^ | 23.58 ± 1.97^a^ | 69.96 ± 2.02^a^ |
| **T_7_**: 75% RDF + *P*. *fluorescens* + *B*. *subtilis* | 40.10 ± 1.12^ab^ | 22.23 ± 0.95^a^ | 62.33 ± 2.08^ab^ | 38.08 ± 1.91^b^ | 21.59 ± 2.27^a^ | 59.67 ± 3.41^bc^ |
| **T_8_**: 75% RDF + *T*. *harzianum* + *B*. *subtilis* | 37.80 ± 4.19^abc^ | 23.19 ± 1.25^a^ | 60.98 ± 4.04^ab^ | 40.10 ± 0.55^ab^ | 22.68 ± 1.21^a^ | 62.77 ± 0.67^b^ |
| **T_9_**: 75% RDF + *T*. *harzianum* + *P*. *fluorescens* + *B*. *subtilis* | 30.05 ± 0.68^bc^ | 21.93 ± 1.55^a^ | 51.98 ± 1.74^b^ | 32.00 ± 3.14^b^ | 22.06 ± 0.97^a^ | 54.06 ± 2.25^c^ |

Different letters indicate significant differences at *P* ≤ 0.05 among the treatments as per DMRT.

**Table S2.** Effect of bio-priming and fertilisation on P uptake by red cabbage

| **Treatments** | **P uptake (kg ha^-1^)** | | | | | |
| --- | --- | --- | --- | --- | --- | --- |
|  | **2016-17** | | | **2017-18** | | |
|  | **Head** | **Stalk** | **Total** | **Head** | **Stalk** | **Total** |
| **T_1_**: Absolute control N:P_2_O_5_:K_2_O @ 0:0:0 kg ha^-1^ | 1.28 ± 0.13^c^ | 2.42 ± 0.05^b^ | 3.69 ± 0.18^c^ | 0.80 ± 0.15^c^ | 2.64 ± 0.14^b^ | 3.44 ± 0.24^d^ |
| **T_2_**: RDF of N:P_2_O_5_:K_2_O @ 120:60:60 kg ha^-1^ | 5.08 ± 0.74^b^ | 3.52 ± 0.22^a^ | 8.60 ± 0.52^b^ | 5.45 ± 0.32^b^ | 3.69 ± 0.14^a^ | 9.14 ± 0.46^bc^ |
| **T_3_**: 75% RDF + *Trichoderma harzianum* | 5.19 ± 0.63^b^ | 3.30 ± 0.14^a^ | 8.50 ± 0.71^b^ | 5.34 ± 0.51^b^ | 3.70 ± 0.13^a^ | 9.04 ± 0.42^bc^ |
| **T_4_**: 75% RDF + *Pseudomonas fluorescens* | 5.76 ± 0.15^ab^ | 3.73 ± 0.36^a^ | 9.49 ± 0.36^ab^ | 6.48 ± 0.35^ab^ | 3.82 ± 0.16^a^ | 10.30 ± 0.22^ab^ |
| **T_5_**: 75% RDF + *Bacillus subtilis* | 5.08 ± 0.28^b^ | 3.69 ± 0.11^a^ | 8.78 ± 0.29^b^ | 5.54 ± 0.23^b^ | 3.40 ± 0.20^a^ | 8.94 ± 0.43^bc^ |
| **T_6_**: 75% RDF + *T*. *harzianum* + *P*. *fluorescens* | 7.36 ± 0.97^a^ | 3.62 ± 0.31^a^ | 10.98 ± 0.66^a^ | 7.52 ± 0.81^a^ | 3.77 ± 0.12^a^ | 11.29 ± 0.70^a^ |
| **T_7_**: 75% RDF + *P*. *fluorescens* + *B*. *subtilis* | 7.09 ± 0.42^a^ | 3.91 ± 0.23^a^ | 10.99 ± 0.39^a^ | 7.32 ± 0.67^a^ | 3.75 ± 0.36^a^ | 11.07 ± 0.89^a^ |
| **T_8_**: 75% RDF + *T*. *harzianum* + *B*. *subtilis* | 6.03 ± 0.76^ab^ | 3.61 ± 0.09^a^ | 9.64 ± 0.67^ab^ | 6.08 ± 0.26^ab^ | 3.59 ± 0.28^a^ | 9.67 ± 0.42^abc^ |
| **T_9_**: 75% RDF + *T*. *harzianum* + *P*. *fluorescens* + *B*. *subtilis* | 4.87 ± 0.16^b^ | 3.38 ± 0.11^a^ | 8.26 ± 0.06^b^ | 4.96 ± 0.52^b^ | 3.59 ± 0.14^a^ | 8.55 ± 0.44^c^ |

Different letters indicate significant differences at *P* ≤ 0.05 among the treatments as per DMRT.

**Table S3.** Effect of bio-priming and fertilisation on K uptake by red cabbage

| **Treatments** | **K uptake (kg ha^-1^)** | | | | | |
| --- | --- | --- | --- | --- | --- | --- |
|  | **2016-17** | | | **2017-18** | | |
|  | **Head** | **Stalk** | **Total** | **Head** | **Stalk** | **Total** |
| **T_1_**: Absolute control N:P_2_O_5_:K_2_O @ 0:0:0 kg ha^-1^ | 12.40 ± 0.78^d^ | 16.64 ± 0.29 | 29.03 ± 1.02^d^ | 8.25 ± 1.84^c^ | 17.84 ± 0.37 | 26.08 ± 1.94^c^ |
| **T_2_**: RDF of N:P_2_O_5_:K_2_O @ 120:60:60 kg ha^-1^ | 43.15 ± 6.38^bc^ | 21.37 ± 0.20 | 64.51 ± 6.51^bc^ | 43.56 ± 3.46^b^ | 21.09 ± 1.34 | 64.65 ± 2.39^b^ |
| **T_3_**: 75% RDF + *Trichoderma harzianum* | 42.34 ± 2.08^bc^ | 20.20 ± 0.45 | 62.53 ± 2.10^c^ | 44.45 ± 4.16^b^ | 21.43 ± 0.41 | 65.88 ± 3.79^b^ |
| **T_4_**: 75% RDF + *Pseudomonas fluorescens* | 44.75 ± 2.93^bc^ | 22.09 ± 2.37 | 66.84 ± 3.04^abc^ | 46.84 ± 0.97^ab^ | 21.92 ± 1.79 | 68.76 ± 1.01^ab^ |
| **T_5_**: 75% RDF + *Bacillus subtilis* | 41.78 ± 2.49^c^ | 22.15 ± 1.73 | 63.93 ± 1.41^bc^ | 44.11 ± 0.99^b^ | 21.46 ± 1.17 | 65.57 ± 1.86^b^ |
| **T_6_**: 75% RDF + *T*. *harzianum* + *P*. *fluorescens* | 57.35 ± 6.17^a^ | 20.86 ± 0.96 | 78.20 ± 6.46^a^ | 56.67 ± 5.07^a^ | 21.84 ± 1.80 | 78.51 ± 6.85^a^ |
| **T_7_**: 75% RDF + *P*. *fluorescens* + *B*. *subtilis* | 54.57 ± 2.08^ab^ | 22.13 ± 0.54 | 76.70 ± 2.61^ab^ | 55.23 ± 3.79^a^ | 21.49 ± 1.81 | 76.72 ± 5.21^ab^ |
| **T_8_**: 75% RDF + *T*. *harzianum* + *B*. *subtilis* | 49.44 ± 5.23^abc^ | 21.81 ± 1.65 | 71.26 ± 6.42^abc^ | 51.34 ± 0.98^ab^ | 21.74 ± 1.42 | 73.09 ± 2.20^ab^ |
| **T_9_**: 75% RDF + *T*. *harzianum* + *P*. *fluorescens* + *B*. *subtilis* | 40.78 ± 0.79^c^ | 20.43 ± 0.22 | 61.21 ± 0.58^c^ | 43.02 ± 4.84^b^ | 21.30 ± 0.80 | 64.32 ± 4.24^b^ |

Different letters indicate significant differences at *P* ≤ 0.05 among the treatments as per DMRT.
